# Health Utility Survival for Randomized Clinical Trials: Extensions and Statistical Properties

**DOI:** 10.1002/sim.70215

**Published:** 2025-08-07

**Authors:** Yangqing Deng, Meiling Hao, Shao Hui Huang, Geoffrey Liu, John R. de Almeida, Wei Xu

**Affiliations:** ^1^ Department of Biostatistics University Health Network Toronto Ontario Canada; ^2^ School of Statistics University of International Business and Economics Beijing China; ^3^ Department of Radiation Oncology University Health Network Toronto Ontario Canada; ^4^ Temerty Faculty of Medicine University of Toronto Toronto Ontario Canada; ^5^ Medical Oncology and Hematology Princess Margaret Cancer Centre Toronto Ontario Canada; ^6^ Dalla Lana School of Public Health University of Toronto Toronto Ontario Canada; ^7^ Department of Otolaryngology—H&N Surgery University Health Network Toronto Ontario Canada; ^8^ Institute of Health Policy, Management and Evaluation University of Toronto Toronto Ontario Canada

**Keywords:** hazard ratio, health utility, overall survival, proportional hazards, randomized controlled trials, time‐to‐event data

## Abstract

Overall survival has been used as the primary endpoint for many randomized trials that aim to examine whether a new treatment is non‐inferior to the standard treatment or placebo control. When a new treatment is indeed non‐inferior in terms of survival, it may be important to assess other outcomes including health utility. However, analyzing health utility scores in a secondary analysis may have limited power since the primary objectives of the original study design may not include health utility. To comprehensively consider both survival and health utility, we developed a composite endpoint, HUS (Health Utility‐adjusted Survival), which combines both survival and utility. HUS has been shown to be able to increase statistical power and potentially reduce the required sample size compared to the standard overall survival endpoint. Nevertheless, the asymptotic properties of the test statistics of the HUS endpoint have yet to be fully established. Besides that, the standard version of HUS cannot be applied to or has limited performance in certain scenarios, where extensions are needed. In this manuscript, we propose various methodological extensions of HUS and derive the asymptotic distributions of the test statistics. By comprehensive simulation studies and a data application using retrospective data based on a translational patient cohort in Princess Margaret Cancer Centre, we demonstrate the better efficiency and feasibility of HUS compared to different methods.

## 1 Introduction

In clinical studies, including superiority and non‐inferiority trials, overall survival (OS) is commonly used as the primary endpoint to compare a new treatment with a standard or controlled treatment. In some scenarios, non‐inferiority trials are preferred due to some specific potential benefits of the new treatment (e.g., lower costs, fewer side effects, better quality of life (QoL), or less difficulty to implement), and it would suffice to show that the new treatment is not worse than control with respect to OS. After establishing non‐inferiority, the next step is usually to demonstrate that the new treatment can benefit patients in some other clinical endpoints beyond OS, and one of such endpoints that clinicians have interest is health utility [1]. Health utility is a value ascribed to individuals' preference for a specific health state. As a measurement usually ranging from 0 to 1, health utility can quantify the health state of a patient at a certain time point, and a higher value usually corresponds to a healthier state. Usually, a health utility of 0 is akin to death and, in some instances, negative utilities can indicate a state worse than death. Health utilities are typically elicited either indirectly from patients by means of patient‐reported outcomes with instruments such as the EQ‐5D or they can be directly measured with utility elicitation techniques such as time‐trade‐off or standard gamble, whereby an individual is presented with a hypothetical health state and asked to select between the hypothetical health state and a lesser time or lower probability of a healthier state, respectively. Various methods for performing statistical analysis using health utility scores at different time points have been proposed in the literature [2, 3, 4]. However, the health utility analysis may be underpowered since the primary objective of the study design is usually based on OS, without taking health utility into consideration, especially if the compared treatments differ substantially in survival but only moderately in utility. In some trials, utility is only evaluated if OS demonstrates a significant improvement. Meanwhile, it may not be desirable to perform statistical testing on OS and health utility separately since it will result in multiplicity and require multiple testing adjustment, which will lead to potential loss of statistical power. Hence, using a composite endpoint that combines survival and health utility to perform a single test may be preferred, since it may increase the statistical power and reduce the required sample size. Furthermore, if utility is analyzed separately, the absence of utility scores following a patient's death constitutes informative missingness. One might assume that utility is zero at and after death; however, this assumption may not always hold and can lead to biased results. Therefore, incorporating both survival and utility into a composite endpoint may naturally offer a more appropriate approach for handling such a missing mechanism.

While methods like Q‐TWiST (Quality‐adjusted Time Without Symptoms of disease or Toxicity) that can combine survival and utility have been proposed and used to analyze clinical trial data, they have their own drawbacks [5, 6, 7, 8, 9, 10, 11, 12]. For instance, though researchers have derived some statistical properties as well as sample size formulas for Q‐TWiST [8], the implementation of this approach is limited to scenarios where each patient's status can be divided into three states (toxicity, time without symptoms and toxicity, and relapse), and the weights for different states are artificially preselected. In many other scenarios, especially with utility scores measured on a continuous scale, clinicians may be more interested in analyzing them in the original scale rather than forcing them into three categories, since some information is lost in the categorization process, which will make the statistical test have lower power.

A more general approach, usually referred to as QALY (quality‐adjusted life years), offers an intuitive way to combine survival and health utility [5, 13, 14, 15, 16, 17], and a similar concept called quality‐adjusted progression‐free survival has also been used in some randomized trials [18, 19, 20]. Nevertheless, the formal statistical frameworks of these methods have not been established, and the potential advantages and feasibility of these methods compared to the traditional survival endpoint have not been fully evaluated through comprehensive simulation studies.

A composite endpoint called HUS (Health Utility‐adjusted Survival) was proposed in recent literature [21], which can combine longitudinal health utility and survival performance to assess treatment effects. The authors also provided a detailed statistical testing framework and procedures for power analysis and sample size calculations. Although they demonstrated through simulations that HUS may increase the statistical power over classic tests based on OS only and thus reduce the required sample size, the asymptotic properties of HUS have yet to be thoroughly explored. Establishing detailed theoretical properties may make HUS a more solid approach that can benefit future clinical trials.

Meanwhile, on some special occasions, it may be beneficial to modify the test statistics in order to better capture treatment effects. For example, the utility scores recorded at later time‐points may be more important than those recorded at earlier time‐points, since they are better indicators of how well a patient has recovered, or ultimately how much the treatment has helped the patients improve their QoL. The early utility scores may indicate a patient's discomfort level during or right after going through a treatment such as surgery, but they may be less important if the clinicians care more about the patient's QoL after the recovering process. In some other scenarios, with multiple measurements of utility recorded at each time‐point, it may be useful to consider giving them different weights before combining them into a single utility score, and the weights may be predetermined based on the clinicians' knowledge.

With the above considerations, we propose some important extensions of HUS, including a time‐weighted version that allows assigning different weights to different time points, denoted by twHUS, and a natural way to combine different utility measurements using weights. We also provide a simple process to apply HUS to clinical observational data with covariates, which may greatly extend the range of studies that HUS can be implemented on. Most importantly, we derive the asymptotic theories for HUS and the proposed extensions.

This manuscript is structured as follows. In Section 2, we present the methodology of the HUS endpoint as well as its extensions, and then we establish the theoretical properties of HUS. In Section 3, we use comprehensive simulation studies, including different scenarios with a single utility score or multiple QoL scores, without covariates or with covariates, to demonstrate the effectiveness of the HUS endpoint. At last, we provide a thorough discussion regarding the strengths and drawbacks of HUS as well as potential future directions in Section 4.

## 2 Methods

### 2.1 Health Utility Survival (HUS)

In this section, we give a brief review of HUS. Suppose the total length of the study is T, and $S_{g}\left(t\right)$ and ${\bar{U}}_{g}\left(t\right)$ are the survival function (proportion of patients alive at t) and average utility score of those alive at t for treatment group g (g=1,2). Intuitively, we can define a composite endpoint using $\int_{0}^{T}S_{1}\left(t\right){\bar{U}}_{1}\left(t\right)\mathrm{dt}$ and $\int_{0}^{T}S_{2}\left(t\right){\bar{U}}_{2}\left(t\right)\mathrm{dt}$. To allow survival and utility to be weighted differently, we propose a highly general class of tests analogous to the two‐sample test proposed in Reference [22], which is defined as

(1) $$Q_{\mathrm{HUS},1}=\int_{0}^{T}{\left[S_{1}\left(t\right)\right]}^{\lambda_{1}}{\left[{\bar{U}}_{1}\left(t\right)\right]}^{\lambda_{2}}\mathrm{dt}$$

(2) $$Q_{\mathrm{HUS},2}=\int_{0}^{T}{\left[S_{2}\left(t\right)\right]}^{\lambda_{1}}{\left[{\bar{U}}_{2}\left(t\right)\right]}^{\lambda_{2}}\mathrm{dt}$$

where $\lambda_{1}$ and $\lambda_{2}$ are preselected weights to reflect the importance of survival and utility. Larger weights correspond to higher importance, and the standard HUS uses $\lambda_{1}=\lambda_{2}=1$. Since true $S_{1}\left(t\right)$ and $S_{2}\left(t\right)$ are unknown, we need to estimate them based on observed data. The simplest way is to obtain Kaplan–Meier (KM) estimates for the two groups separately [23]. Then we can substitute $S_{1}\left(t\right)$ and $S_{2}\left(t\right)$ with ${\hat{S}}_{1}\left(t\right)$ and ${\hat{S}}_{2}\left(t\right)$.

We may also use the Cox proportional hazards model [24], treating the treatment assignment as a covariate, to obtain estimates of the survival functions. This requires the proportional hazards assumption for the two groups, which may have benefits when this assumption is not violated.

The test statistic to examine whether the two treatment groups differ in HUS is defined as

(3) $$T=Q_{\mathrm{HUS},1}-Q_{\mathrm{HUS},2}$$

We can use the bootstrap or the permutation algorithm to get the empirical confidence intervals or *p*‐values. With our derived asymptotic properties of HUS, we may also use an alternative approach to simulate the distribution of T and obtain its *p*‐value. More details are provided in Appendix A in Data S1.

### 2.2 Time‐Weighted HUS (twHUS)

In some clinical studies, the importance of health utility at different time points may vary. For example, the utility scores recorded in the later stage of the study may be more important than those recorded in the earlier stage (e.g., around surgery time), as the later scores may show how well a patient has recovered. Besides, some treatments may show less effect in the beginning, but benefit the patients' QoL significantly more after a period of time. As a result, it may make more sense to give different weights to utility scores at different time points. We propose a twHUS, with

(4) $$Q_{\text{tHUS},1}=\int_{0}^{T}{\left[S_{1}\left(t\right)\right]}^{\lambda_{1}}{\left[{\bar{U}}_{1}\left(t\right)w\left(t\right)\right]}^{\lambda_{2}}\mathrm{dt}$$

(5) $$Q_{\text{tHUS},2}=\int_{0}^{T}{\left[S_{2}\left(t\right)\right]}^{\lambda_{1}}{\left[{\bar{U}}_{2}\left(t\right)w\left(t\right)\right]}^{\lambda_{2}}\mathrm{dt}$$

where w(t) is a function of weight across time. For example, we may let w(t) linearly increase from 0 at baseline to 1 at the end of surgery, and then it may stay at 1 until the end of study. We use this setting by default unless otherwise specified.

### 2.3 Special Case With Multiple QoL Scores

Note that our previous framework only focuses on one utility score, while sometimes we may have different QoL scores measuring different aspects of the wealth and comfort of patients. Suppose we have M different QoL scores, and $U_{\mathrm{gi},m}\left(t\right)$ is the mth QoL score for subject i at time t in treatment group g (g=1,2). To combine different scores, we may define a new score $U_{\mathrm{gi}}^{*}$, with

(6) $$U_{\mathrm{gi}}^{*}\left(t\right)=\sum_{m=1}^{M}v_{m}U_{\mathrm{gi},m}\left(t\right)$$

where ${v_{m}}^{\prime }s$ are the weights for different scores, and $\sum_{m=1}^{M}v_{m}=1$. In some cases, if we are interested in the worst score at each time point, we may also consider $U_{\mathrm{gi}}^{*}\left(t\right)=\min_{m}\left(U_{\mathrm{gi},m}\left(t\right)\right)$. Once the combined score is defined and calculated, we can apply our previously described procedures.

### 2.4 HUS For Observational Data

The original framework of HUS was intended for data from randomized trials, where covariates are balanced in the two treatment groups. However, we may also be interested in analyzing observational studies that the study subjects were not randomized by treatments. In this case, due to the potential confounders that are likely unbalanced, directly applying HUS to the full data may be problematic. A simple but effective approach to alleviate the confounding issue is to conduct propensity matching first, and then analyze the matched pairs [25]. While there are more sophisticated techniques regarding propensity scores in survival analyses [26, 27, 28], to maintain simplicity and focus, we will only demonstrate propensity score matching in our simulations.

### 2.5 Theoretical Properties

#### 2.5.1 Asymptotic Distribution of HUS

In this section, we investigate the asymptotic properties of the HUS test statistic. The observed test statistic is $\hat{T}={\hat{Q}}_{\mathrm{HUS},1}-{\hat{Q}}_{\mathrm{HUS},2}$, with

(7) $${\hat{Q}}_{\mathrm{HUS},1}=\int_{0}^{T}{\left[{\hat{S}}_{1}\left(t\right)\right]}^{\lambda_{1}}{\left[{\bar{U}}_{1}\left(t\right)\right]}^{\lambda_{2}}\mathrm{dt}$$

(8) $${\hat{Q}}_{\mathrm{HUS},2}=\int_{0}^{T}{\left[{\hat{S}}_{2}\left(t\right)\right]}^{\lambda_{1}}{\left[{\bar{U}}_{2}\left(t\right)\right]}^{\lambda_{2}}\mathrm{dt}$$

For the simplest case without weights, we have $\lambda_{1}=\lambda_{2}=1$, and

(9) $${\hat{Q}}_{\mathrm{HUS},1}=\int_{0}^{T}{\hat{S}}_{1}\left(t\right){\bar{U}}_{1}\left(t\right)\mathrm{dt}$$

(10) $${\hat{Q}}_{\mathrm{HUS},2}=\int_{0}^{T}{\hat{S}}_{2}\left(t\right){\bar{U}}_{2}\left(t\right)\mathrm{dt}$$

Note that in the above formulas, we have already replaced the unknown survival functions with KM estimates ${\hat{S}}_{1}\left(t\right)$ and ${\hat{S}}_{2}\left(t\right)$. Denote the survival time, censoring time, and observed time of the ith subject in group g by $T_{\mathrm{gi}}$, $C_{\mathrm{gi}}$, and $X_{\mathrm{gi}}$ respectively, and their utility score at time t by $U_{\mathrm{gi}}\left(t\right)$, of which the expectation is $U_{g}\left(t\right)$. Define $N_{\mathrm{gi}}\left(t\right)=I\left\{X_{\mathrm{gi}}\leq t,{∆}_{\mathrm{gi}}=1\right\}$, ${∆}_{\mathrm{gi}}=I\left\{X_{\mathrm{gi}}\leq C_{\mathrm{gi}}\right\}$, $Y_{\mathrm{gi}}\left(t\right)=I\left\{X_{\mathrm{gi}}\geq t\right\}$, ${\bar{Y}}_{g}\left(t\right)=\sum_{i=1}^{n_{g}}Y_{\mathrm{gi}}\left(t\right)$, g=1,2.

The following assumptions are needed to establish the asymptotic distribution of T under the null hypothesis.

Assumption A.1 $P\left(T_{\mathrm{gi}}>T\right)>0.$

Assumption A.2 $U_{1}\left(t\right),U_{2}\left(t\right)$ are of bounded variation on [0,T].

Assumption A.1 is very common in survival analysis [29, 30]. Assumption A.2 is to make sure the weak convergence of $\sqrt{n_{g}}\left\{{\bar{U}}_{g}\left(t\right)-U_{\mathrm{gi}}\left(t\right)\right\}$, g=1,2, which is very common in counting process theory.

Lemma 1 *Under Assumptions* *A.1* *and* *A.2*, *we have* $\sqrt{n_{1}}\left\{{\hat{S}}_{1}\left(t\right){\bar{U}}_{1}\left(t\right)-S_{1}\left(t\right)U_{1}\left(t\right)\right\}$ *converges to a mean zero Gaussian process* $G_{1}\left(t\right)U_{1}\left(t\right)+S_{1}\left(t\right)U_{1}\left(t\right)$. *Here*, $G_{1}\left(t\right)$ *is a mean zero Gaussian process with variance being* $S_{1}^{2}\left(t\right)\int_{0}^{t}\lambda \left(u\right)/p\left(X_{1i}>u\right)\mathrm{du}$, $U_{1}\left(t\right)$ *is a mean zero Gaussian process with variance being* $E{\left[U_{1i}\left(t\right)-U_{1}\left(t\right)\right]}^{2}$, *and* λ(t) *is the hazard function for* $X_{1i}$.

Theorem 1 *Under Assumptions* *A.1* *and* *A.2* *and the null hypothesis, if* $\frac{n_{1}}{n_{2}}\to c>0$, *we have*

(11) $$\sqrt{n_{1}}\hat{T}\to_{d}\sqrt{1+c}\int_{0}^{T}\;G_{1}\left(t\right)U_{1}\left(t\right)+S_{1}\left(t\right)U_{1}\left(t\right)\mathrm{dt}$$

*Here*, $\to_{d}$ *means converges in distribution. More details and the proofs of Lemma* *1* *and Theorem 1 are provided in Appendix* A *of Data* S1.

We can derive asymptotic properties using similar techniques for HUS with weights, where $\lambda_{1},\lambda_{2}$ are preselected values.

Lemma 2 *Under Assumptions* *A.1* *and* *A.2*, *we have* $\sqrt{n_{1}}\left\{{\left[{\hat{S}}_{1}\left(t\right)\right]}^{\lambda_{1}}{\left[{\bar{U}}_{1}\left(t\right)\right]}^{\lambda_{2}}-{\left[S_{1}\left(t\right)\right]}^{\lambda_{1}}{\left[U_{1}\left(t\right)\right]}^{\lambda_{2}}\right\}$ *converges to a mean zero Gaussian process*:

$$\lambda_{1}G_{1}\left(t\right){\left[S_{1}\left(t\right)\right]}^{\lambda_{1}-1}{\left[U_{1}\left(t\right)\right]}^{\lambda_{2}}+\lambda_{2}{\left[S_{1}\left(t\right)\right]}^{\lambda_{1}}{\left[U_{1}\left(t\right)\right]}^{\lambda_{2}-1}U_{1}\left(t\right)$$

Theorem 2 *Under Assumptions* *A.1* *and* *A.2* *and the null hypothesis, if* $\frac{n_{1}}{n_{2}}\to c$, *we have*

(12) $${\sqrt{n}}_{1}\hat{T}\to_{d}\sqrt{1+c}\int_{0}^{T}\;\lambda_{1}G_{1}\left(t\right){\left[S_{1}\left(t\right)\right]}^{\lambda_{1}-1}{\left[U_{1}\left(t\right)\right]}^{\lambda_{2}}+\lambda_{2}{\left[S_{1}\left(t\right)\right]}^{\lambda_{1}}{\left[U_{1}\left(t\right)\right]}^{\lambda_{2}-1}U_{1}\left(t\right)dt$$

The proofs for Lemma 2 and Theorem 2 are similar to the proofs for Lemma 1 and Theorem 1. They are available in Appendix A of Data S1. Under the null hypothesis, the distribution of $\sqrt{n_{1}}\hat{T}$ can be approximated well via a perturbation‐resampling method [31, 32, 33]. Let $Z_{1i}\left(i=1,\ldots ,n_{1}\right)$ and $Z_{2i}\left(i=1,\ldots ,n_{2}\right)$ be independent random samples from N(0,1). For HUS with weights $\lambda_{1},\lambda_{2}$, following from the proof of Lemma 2, we can approximate the distribution of $\sqrt{n_{1}}\hat{T}$ by

(13) $$\begin{array}{ll} \Gamma^{*} & =\sqrt{n_{1}}\int_{0}^{T\;}\lambda_{1}{\hat{S}}_{1}^{\lambda_{1}-1}\left(t\right){\hat{S}}_{1}\left(t\right)\sum_{i=1}^{n_{1}}Z_{1i}\int_{0}^{t}\frac{I\left\{X_{1i}\leq t,\Delta_{1i}=1\right\}}{{\bar{Y}}_{1}\left(X_{1i}\right)}{\bar{U}}_{1}^{\lambda_{2}}\left(t\right)\mathrm{dt}\\ & \;+\sqrt{n_{1}^{-1}}\int_{0}^{T\;}\lambda_{2}{\hat{S}}_{1}^{\lambda_{1}}\left(t\right){\bar{U}}_{1}^{\lambda_{2}-1}\left(t\right)\sum_{i=1}^{n_{1}}Z_{1i}\left\{U_{1i}\left(t\right)-{\bar{U}}_{1}\left(t\right)\right\}\mathrm{dt}\\ & \;-\sqrt{\frac{n_{1}}{n_{2}}}\{\sqrt{n_{2}}\int_{0}^{T\;}\lambda_{1}{\hat{S}}_{2}^{\lambda_{1}-1}\left(t\right){\hat{S}}_{2}\left(t\right)\sum_{i=1}^{n_{1}}Z_{2i}\int_{0}^{t}\frac{I\left\{X_{2i}\leq t,\Delta_{2i}=1\right\}}{{\bar{Y}}_{2}\left(X_{2i}\right)}{\bar{U}}_{2}^{\lambda_{2}}\left(t\right)\mathrm{dt}\\ & \;+\sqrt{n_{2}^{-1}}\int_{0}^{T}\lambda_{2}{\hat{S}}_{2}^{\lambda_{1}}\left(t\right){\bar{U}}_{2}^{\lambda_{2}-1}\left(t\right)\sum_{i=1}^{n_{1}}Z_{2i}\left\{U_{2i}\left(t\right)-{\bar{U}}_{2}\left(t\right)\right\}\mathrm{dt}\} \end{array}$$

According to our experience, this approximation approach has similar performance compared to the bootstrap method. As the bootstrap method is more straightforward and more commonly used, we recommend using it by default. The results in this manuscript are based on bootstrap, unless otherwise specified. Some comparison of the two methods can be found in Appendix B of Data S1.

#### 2.5.2 Asymptotic Distribution of Time‐Weighted HUS

For twHUS, the test statistics are calculated using

(14) $${\hat{Q}}_{\text{tHUS},1}=\int_{0}^{T}{\left[{\hat{S}}_{1}\left(t\right)\right]}^{\lambda_{1}}{\left[{\bar{U}}_{1}\left(t\right)w\left(t\right)\right]}^{\lambda_{2}}\mathrm{dt}$$

(15) $${\hat{Q}}_{\text{tHUS},2}=\int_{0}^{T}{\left[{\hat{S}}_{2}\left(t\right)\right]}^{\lambda_{1}}{\left[{\bar{U}}_{2}\left(t\right)w\left(t\right)\right]}^{\lambda_{2}}\mathrm{dt}$$

To compare two treatment arms, we look at ${\hat{T}}_{t}={\hat{Q}}_{\text{tHUS},1}-{\hat{Q}}_{\text{tHUS},2}$. Here ${\hat{T}}_{t}$ is defined as the test statistic, and we want to give the asymptotic distribution under the null hypothesis.

Lemma 3 *Under Assumptions* *A.1* *and* *A.2*, *we have* $\sqrt{n_{1}}\left\{{\left[{\hat{S}}_{1}\left(t\right)\right]}^{\lambda_{1}}{\left[w\left(t\right){\bar{U}}_{1}\left(t\right)\right]}^{\lambda_{2}}-{\left[S_{1}\left(t\right)\right]}^{\lambda_{1}}{\left[w\left(t\right)U_{1}\left(t\right)\right]}^{\lambda_{2}}\right\}$ *converges to a mean zero Gaussian process*, $\lambda_{1}G_{1}\left(t\right){\left[S_{1}\left(t\right)\right]}^{\lambda_{1}-1}{\left[w\left(t\right)U_{1}\left(t\right)\right]}^{\lambda_{2}}+\lambda_{2}{\left[S_{1}\left(t\right)\right]}^{\lambda_{1}}{\left[w\left(t\right)\right]}^{\lambda_{2}}{\left[U_{1}\left(t\right)\right]}^{\lambda_{2}-1}U_{1}\left(t\right)$.

Theorem 3 *Under Assumptions* *A.1* *and* *A.2* *and the null hypothesis, if* $\frac{n_{1}}{n_{2}}\to c$, *we have*

(16) $$\begin{array}{cc} & \sqrt{n}{\hat{T}}_{t}\to_{d}\sqrt{1+c}\int_{0}^{T}\;\lambda_{1}G_{1}\left(t\right){\left[S_{1}\left(t\right)\right]}^{\lambda_{1}-1}{\left[w\left(t\right)U_{1}\left(t\right)\right]}^{\lambda_{2}}\\ & \;+\lambda_{2}{\left[S_{1}\left(t\right)\right]}^{\lambda_{1}}{\left[w\left(t\right)\right]}^{\lambda_{2}}{\left[U_{1}\left(t\right)\right]}^{\lambda_{2}-1}U_{1}\left(t\right)\mathrm{dt} \end{array}$$

The proofs for Lemma 3 and Theorem 3 are provided in Appendix A of Data S1. For twHUS, by perturbation‐resampling [31, 32, 33], it is also possible to approximate the distribution of $\sqrt{n_{1}}{\hat{T}}_{t}$ by

(17) $$\begin{array}{ll} \Gamma_{t}^{*} & =\sqrt{n_{1}}\int_{0}^{T\;}\lambda_{1}{\hat{S}}_{1}^{\lambda_{1}-1}\left(t\right){\hat{S}}_{1}\left(t\right)\sum_{i=1}^{n_{1}}Z_{1i}\int_{0}^{t}\frac{I\left\{X_{1i}\leq t,\Delta_{1i}=1\right\}}{{\bar{Y}}_{1}\left(X_{1i}\right)}\\ & \;\times {\left[w\left(t\right){\bar{U}}_{1}\left(t\right)\right]}^{\lambda_{2}}\mathrm{dt}+\sqrt{n_{1}^{-1}}\int_{0}^{T}\lambda_{2}{\hat{S}}_{1}^{\lambda_{1}}\left(t\right){\left[w\left(t\right)\right]}^{\lambda_{2}}{\bar{U}}_{1}^{\lambda_{2}-1}\left(t\right)\\ & \;\times \sum_{i=1}^{n_{1}}Z_{1i}\left\{U_{1i}\left(t\right)-{\bar{U}}_{1}\left(t\right)\right\}\mathrm{dt}\\ & \;-\sqrt{\frac{n_{1}}{n_{2}}}\{\sqrt{n_{2}}\int_{0}^{T}\lambda_{1}{\hat{S}}_{2}^{\lambda_{1}-1}\left(t\right){\hat{S}}_{2}\left(t\right)\sum_{i=1}^{n_{1}}Z_{2i}\int_{0}^{t}\frac{I\left\{X_{2i}\leq t,\Delta_{2i}=1\right\}}{{\bar{Y}}_{2}\left(X_{2i}\right)}\\ & \;\times {\left[w\left(t\right){\bar{U}}_{2}\left(t\right)\right]}^{\lambda_{2}}\mathrm{dt}+\sqrt{n_{2}^{-1}}\int_{0}^{T}\lambda_{2}{\hat{S}}_{2}^{\lambda_{1}}\left(t\right){\left[w\left(t\right)\right]}^{\lambda_{2}}{\bar{U}}_{2}^{\lambda_{2}-1}\left(t\right)\\ & \;\times \sum_{i=1}^{n_{1}}Z_{2i}\left\{U_{2i}\left(t\right)-{\bar{U}}_{2}\left(t\right)\right\}\mathrm{dt}\} \end{array}$$

### 2.6 Handling Missing Utility Scores

In most of the clinical studies, it is difficult to collect utility scores at every time point for all subjects. As previous literature has shown [21], in scenarios with missing utility scores, a simple but efficient way is to use linear functions to fill in the utility scores for each subject. However, using this approach may result in very unstable estimates when the number of recorded scores for a subject is too small. In this manuscript, we apply an alternative method, where we use the average score of the treatment group at a time point plus a small variation to impute the missing scores at that time point. The small variation follows a normal distribution with its mean equal to zero and variance equal to the sample variance of the non‐missing utility scores at that time point. We only implement this procedure on time points where at least 80% of the subjects have utility scores recorded. Next, we fill in the rest of the missing scores using linear functions for each subject separately. Following this procedure, a single imputation is conducted. Our experience shows that the new imputation method yields higher statistical power compared to the previous approach. Some examples are provided in Appendix B of Data S1.

## 3 Results

### 3.1 Simulations With Single Utility Score

We conduct simulations under various scenarios focusing on comparing the performances of different versions of HUS, as the advantages of the standard version of HUS has already been thoroughly studied [21]. By default, we simulate a randomized clinical trial data with two treatment arms mimicking the PET‐NECK trial, a randomized phase III non‐inferiority trial that compares positron emission tomography‐computerized tomography‐guided watch‐and‐wait policy (PET‐CT) with planned neck dissection (planned ND) for head and neck cancer patients [1]. We assume the length of study to be 36 months (T=36) with each patient receiving surgery at 3 months (C=3). Let $T_{\mathrm{gi}}$, $X_{\mathrm{gi}}$, and $\delta_{\mathrm{gi}}$ be the true survival time, observed survival time, and survival status for patient i from treatment group g, respectively, and $n_{1},n_{2}$ be the sample sizes for group 1 and group 2. Similar to what was done in prior work [21], we simulate the survival data using

$$T_{\mathrm{gi}}∼\mathrm{Exp}\left(h_{g}\right)$$

$$C_{\mathrm{gi}}∼\text{Unif}\left(0,\zeta \right)$$

$$X_{\mathrm{gi}}=\min\left(T_{\mathrm{gi}},C_{\mathrm{gi}},T\right)$$

$$\delta_{\mathrm{gi}}=\left\{\begin{array}{ll} 1 & \left(X_{\mathrm{gi}}<C_{\mathrm{gi}}\;\text{and}\;X_{\mathrm{gi}}<T\right)\\ 0 & \left(\text{otherwise}\right) \end{array}\right.$$

where ζ is chosen to control the censoring rate, denoted by $p_{\text{censoring}}$. Under this setting, it is easy to see that the hazard ratio of treatment 1 against treatment 2 is $h_{1}/h_{2}$. Note that our focus is to compare the performances of different HUS methods with different patterns of the utility difference given that the two treatments do not differ in OS, we let $h_{1}/h_{2}=1$ unless otherwise specified.

**FIGURE 1:**
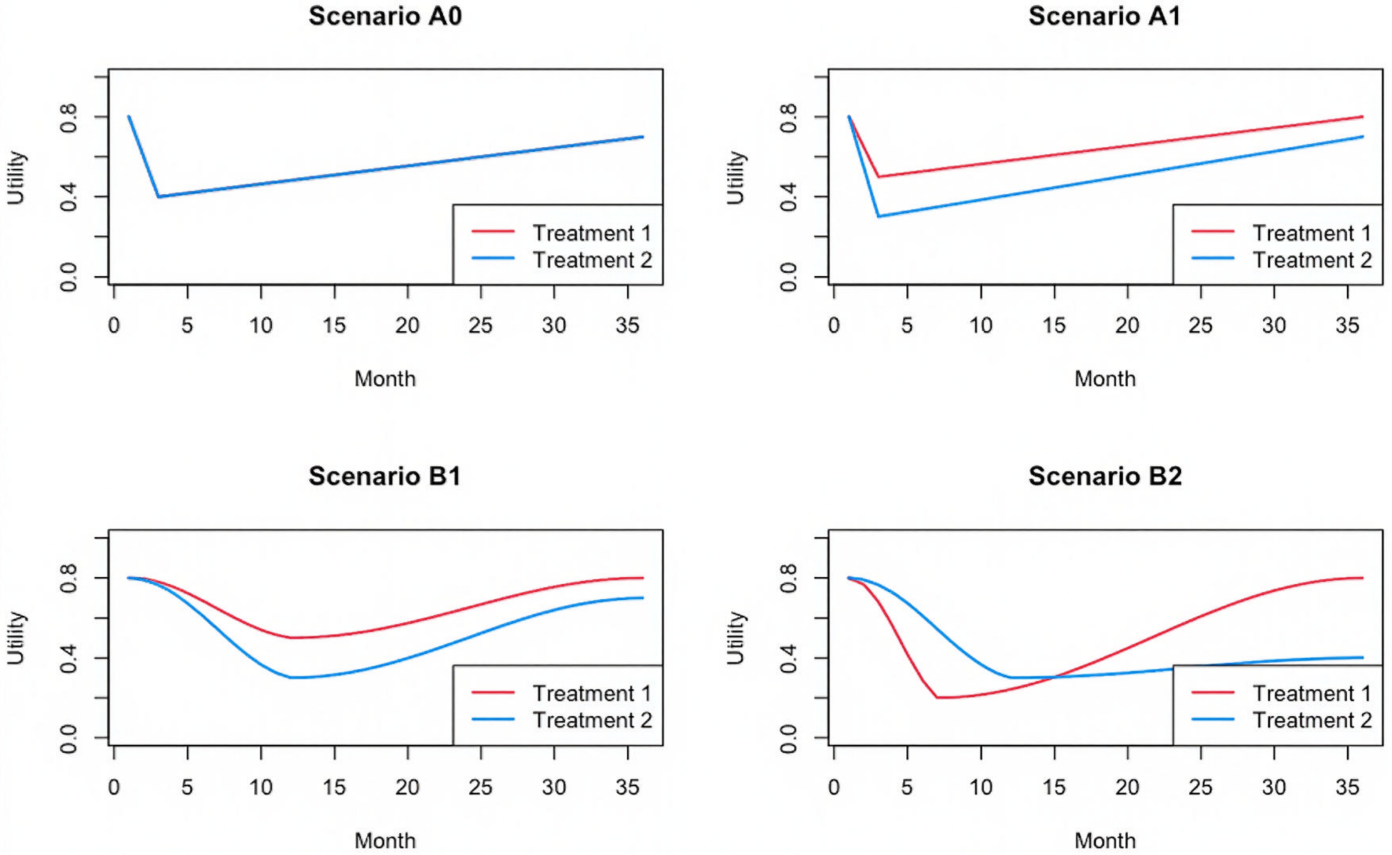
Utility plots for different scenarios with a single utility score.

When simulating the health utility score, we first define the base utility at time t for group g using functions $U_{g0}\left(t\right)$. For example, $U_{g0}\left(t\right)$ can be defined as

$$U_{g0}\left(t\right)=\left\{\begin{array}{ll} A_{g1}+\frac{A_{g2}-A_{g1}}{C}t, & \left(0\leq t\leq C\right);\\ \frac{TA_{g2}-CA_{g3}}{T-C}+\frac{A_{g3}-A_{g2}}{T-C}t, & \left(C<t\leq T\right) \end{array}\right.$$

which represents the average utility for group g starts from $A_{g1}$ at baseline, linearly changes to $A_{g2}$ at 3 months, and then linearly changes to $A_{g3}$ at the end of the study. This setting mimics typical clinical trials where a patient's health utility reaches the lowest at the end of surgery and gradually recovers afterwards. We use $N\left(U_{g0}\left(t\right),0.01\right)$ to generate $U_{\mathrm{gi}}\left(t\right)$, the health utility score of patient i from group g at time‐point t. Since it rarely happens in practice that utility scores are fully collected at all time‐points, we assume that the scores are only collected at t=1, C, and T unless otherwise specified. Also, we assume there is a $p_{\text{missingU}}$ chance that the score is missing for a subject when t=C or T. We choose $p_{\text{missingU}}=0.3$ by default. Figure 1 shows the base utility functions for different scenarios. Note that in Scenarios B1 and B2, the utility changes are piece‐wise smooth but not piece‐wise linear. In Scenario B2, treatment 1 reaches the lowest point earlier than treatment 2. We conducted 200 iterations to assess statistical power in scenarios where the null hypothesis is false and increase the number of iterations to 500 when estimating type I error rates in scenarios where the null hypothesis is true.

As shown in Table 1, after applying different methods to our simulated data, we find that all methods can control type I errors in Scenario A0. Since the difference between the two treatment groups lies in utility but not in survival, assigning more weight to utility results in higher power in Scenarios A1 and B1–B2. Meanwhile, twHUS has similar performance to the standard HUS except in Scenario B2, which makes sense because, in the other scenarios, the difference between the treatments is relatively consistent throughout the study, whereas in Scenario B2, treatment 1 performs worse in the earlier stage but becomes much better in the later stage. In such a scenario, using the time weights with more weights given to the later time points, it is easier to detect the advantage of treatment 1 over that of treatment 2, and thus twHUS can obtain higher power compared to the standard HUS. For both HUS and twHUS, since the survival data was generated under the proportional hazards assumption, the Cox model is able to get slightly better results, and thus yields slightly higher power compared to the KM method. These simulation results demonstrate the flexibility of HUS and that it is important to choose appropriate weights given their potential impact on the testing results. Note that in our main simulations, the missingness of the utility score is independent of its value. In Appendix B of Data S1, we demonstrate that HUS can work relatively well when there is moderate informative missingness, where lower utility scores are more likely to be missing.

**TABLE 1:** Simulation results for Scenarios A0–A1 and B1–B2.

	KM				Cox			
*n*_1_, *n*_2_	*λ*_2_ = 1	*λ*_2_ = 0.5	*λ*_2_ = 2	*λ*_2_ = 1 (twHUS)	*λ*_2_ = 1	*λ*_2_ = 0.5	*λ*_2_ = 2	*λ*_2_ = 1 (twHUS)
Scenario A0								
50	0.048	0.047	0.053	0.048	0.047	0.046	0.052	0.046
100	0.046	0.045	0.051	0.046	0.051	0.048	0.053	0.052
150	0.054	0.054	0.052	0.050	0.052	0.051	0.054	0.052
Scenario A1								
50	0.81	0.41	0.99	0.80	0.85	0.46	1	0.85
100	0.95	0.62	1	0.95	0.98	0.70	1	0.98
150	0.99	0.78	1	0.99	1	0.86	1	1
Scenario B1								
50	0.74	0.32	0.97	0.72	0.80	0.39	0.98	0.78
100	0.90	0.57	1	0.9	0.92	0.60	1	0.92
150	0.99	0.67	1	0.98	0.99	0.76	1	0.98
Scenario B2								
50	0.24	0.11	0.71	0.58	0.30	0.12	0.75	0.62
100	0.44	0.17	0.90	0.82	0.49	0.18	0.92	0.85
150	0.54	0.21	0.99	0.92	0.60	0.22	0.98	0.95

*Note*: Type I errors for Scenario A0, and power for Scenarios A1 and B1–B2.

### 3.2 Simulations With Multiple QoL Scores

**FIGURE 2:**
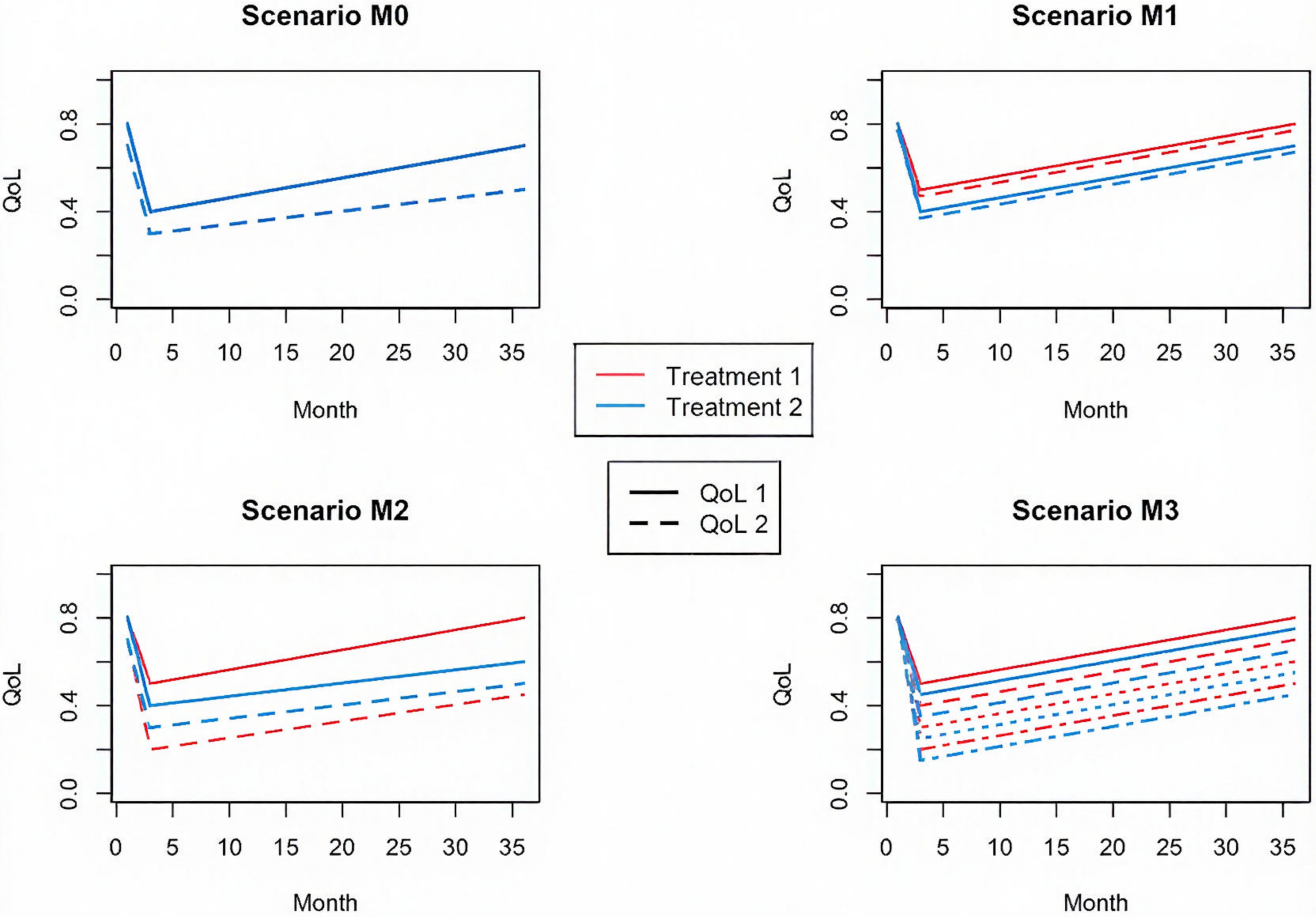
Quality of life plots for different scenarios with multiple measures of quality of life.

To examine the performance of our method for combing multiple measures with weights, we simulate multiple QoL scores using similar procedures as we used in Section 3.1. Figure 2 shows the base QoL functions for different scores in four different scenarios. As shown in Table 2, in Scenario M0, where there is no difference between the two treatments in either measurement, HUS can control the type I error regardless of the choice of weights. In Scenario M1, where the difference is the same in both scores, the choice of weights does not affect the power. In Scenario M2, treatment 1 performs better than treatment 2 in terms of score 1, but it is worse in score 2. As a result, assigning more weight to score 1 (i.e., choosing a larger $v_{1}$) leads to higher power. In Scenario M3, with four QoL scores having the same difference, different choices of weights yield very similar results. This shows that choosing different weights may have a significant impact on the testing result when the differences in scores are not the same. We recommend choosing the same weight for all measurements if there is no prior knowledge of which measurements are more important.

**TABLE 2:** Simulation results for Scenarios M0–M3.

*n*_1_, *n*_2_	$v_{1}=0.5$, $v_{2}=0.5$	$v_{1}=0.8$, $v_{2}=0.2$	$v_{1}=0.2$, $v_{2}=0.8$
Scenario M0			
50	0.050	0.053	0.052
100	0.050	0.051	0.049
150	0.052	0.045	0.051
Scenario M1			
50	0.35	0.33	0.32
100	0.59	0.54	0.54
150	0.69	0.67	0.69
Scenario M2			
50	0.06	0.25	0.02
100	0.12	0.47	0
150	0.14	0.60	0.02

*n*_1_, *n*_2_	$v_{1}=0.25$, $v_{2},v_{3},v_{4}=0.25$	$v_{1}=0.4$, $v_{2},v_{3},v_{4}=0.2$
Scenario M3		
50	0.16	0.14
100	0.32	0.28
150	0.34	0.33

*Note*: Type I errors for Scenario M0, and power for Scenarios M1–M3.

### 3.3 Simulation With Covariates

In this section, we consider scenarios where we have observational data and need to be adjusted for covariates. We simulate 10 000 samples with covariates:

$${\mathrm{Age}}_{i}∼\text{Unif}\left(35,80\right)$$

$${\mathrm{Sex}}_{i}∼\text{Bernoulli}\left(0.5\right)$$

Sex is defined as 0 for female and 1 for male. Each subject is assigned to a treatment group (1 or 2) with a probability that is a function of covariates. The probability of receiving treatment 2 is

$$p_{i}=0.5+\eta_{\mathrm{age}}\left({\mathrm{Age}}_{i}-57.5\right)+\eta_{\mathrm{sex}}\left({\mathrm{Sex}}_{i}-0.5\right)$$

We choose $\eta_{\mathrm{age}}=0.01$ and $\eta_{\mathrm{sex}}=0.2$, meaning that older patients and male patients are more likely to receive treatment 2 (control). As a result, treatment 1 has 60% female and 40% male, while treatment 2 has 40% female and 60% male. The average age is 48.8 for treatment 1 and 56.0 for treatment 2.

For each replication, we randomly select $n_{1}$ subjects from treatment group 1 and $n_{2}$ subjects from treatment group 2. For the ith subject in group g, denote their age and sex by ${\mathrm{Age}}_{\mathrm{gi}}$ and ${\mathrm{Sex}}_{\mathrm{gi}}$ respectively. We simulate the survival data using

$$T_{\mathrm{gi}}∼\mathrm{Exp}\left(h_{g}e^{\beta_{\mathrm{age}}\left({\mathrm{Age}}_{\mathrm{gi}}-57.5\right)+\beta_{\mathrm{sex}}\left({\mathrm{Sex}}_{\mathrm{gi}}-0.5\right)}\right)$$

$$C_{\mathrm{gi}}∼\text{Unif}\left(0,\zeta \right)$$

$$X_{\mathrm{gi}}=\min\left(T_{\mathrm{gi}},\xi_{\mathrm{gi}},T\right)$$

$$\delta_{\mathrm{gi}}=\left\{\begin{array}{ll} 1 & \left(X_{\mathrm{gi}}<C_{\mathrm{gi}}\;\text{and}\;X_{\mathrm{gi}}<T\right)\\ 0 & \left(\text{otherwise}\right) \end{array}\right.$$

where ζ is chosen to control the censoring rate, denoted by $p_{\text{censoring}}$. Given the distributions used to generate age and sex in the full sample, their mathematical expectations are 57.5 and 0.5, respectively. $h_{g}$ represents the baseline hazard rate for group g when age and sex are centered at 57.5 and 0.5. The hazard ratio of treatment 1 against treatment 2, given the same age and sex, is $h_{1}/h_{2}$. The hazard ratio of male versus female given the same age and treatment is $e^{\beta_{\mathrm{sex}}}$ (e.g., this hazard ratio is 1.22 if we choose $\beta_{\mathrm{sex}}=0.2$). The hazard ratio of age 80 versus age 35 given the same sex and treatment is $e^{45\beta_{\mathrm{age}}}$ (e.g., this hazard ratio is 6.05 if we choose $\beta_{\mathrm{age}}=0.04$).

For health utility, we use the same baseline functions $U_{g0}\left(t\right)$ as before. For the ith subject in group g, we simulate its utility scores as

$$U_{\mathrm{gi}}\left(t\right)=U_{g0}\left(t\right)+\beta_{g,\mathrm{age}}\left({\mathrm{Age}}_{\mathrm{gi}}-57.5\right)+\beta_{g,\mathrm{sex}}\left({\mathrm{Sex}}_{\mathrm{gi}}-0.5\right)+e_{\mathrm{gi}}$$

which means we allow the age and sex's effects on health utility to be different depending on the treatment. For example, if we let $\beta_{1,\mathrm{age}}=\beta_{2,\mathrm{age}}=-0.01$ and $\beta_{1,\mathrm{sex}}=\beta_{2,\mathrm{sex}}=-0.1$, it will mean that older patients and male patients tend to have lower utility scores. We consider five scenarios: Scenario C0 uses the same base utility function as in Scenario A0, while Scenarios C1–C4 use the same base utility function as in Scenario A1. The effect sizes of the covariates are included in Table 3.

**TABLE 3:** Effect sizes of covariates and testing results for Scenarios C0–C4.

**Scenario C0**				
Effect size	Assign	Survival	Utility (group 1)	Utility (group 2)
Age	0.01	0.04	−0.01	−0.01
Sex	0.2	0.2	−0.1	−0.1

**Type I error**						
	Naïve			Propensity matching		
*n*_1_, *n*_2_	*λ*_2_ = 1	*λ*_2_ = 0.5	*λ*_2_ = 2	*λ*_2_ = 1	*λ*_2_ = 0.5	*λ*_2_ = 2
50	0.55	0.42	0.62	0.04	0.04	0.03
100	0.84	0.71	0.89	0.04	0.04	0.04

**Scenario C1**				
Effect size	Assign	Survival	Utility (group 1)	Utility (group 2)
Age	0.01	0.04	−0.01	−0.01
Sex	0.2	0.2	−0.1	−0.1

**Power**						
	Naïve			Propensity matching		
*n*_1_, *n*_2_	*λ*_2_ = 1	*λ*_2_ = 0.5	*λ*_2_ = 2	*λ*_2_ = 1	*λ*_2_ = 0.5	*λ*_2_ = 2
50	0.97	0.80	1	0.32	0.21	0.52

**Scenario C2**				
Effect size	Assign	Survival	Utility (group 1)	Utility (group 2)
Age	0.01	−0.02	0.01	0.01
Sex	0.2	−0.1	0.1	0.1

**Power**						
	Naïve			Propensity matching		
*n*_1_, *n*_2_	*λ*_2_ = 1	*λ*_2_ = 0.5	*λ*_2_ = 2	*λ*_2_ = 1	*λ*_2_ = 0.5	*λ*_2_ = 2
50	0.07	0.06	0.10	0.38	0.20	0.52
100	0.07	0.04	0.14	0.63	0.38	0.84
150	0.06	0.03	0.14	0.76	0.44	0.92

**Scenario C3**				
Effect size	Assign	Survival	Utility (group 1)	Utility (group 2)
Age	0	0.04	−0.01	−0.01
Sex	0	0.2	−0.1	−0.1

**Power**						
	Naïve			Propensity matching		
*n*_1_, *n*_2_	*λ*_2_ = 1	*λ*_2_ = 0.5	*λ*_2_ = 2	*λ*_2_ = 1	*λ*_2_ = 0.5	*λ*_2_ = 2
50	0.46	0.24	0.68	0.41	0.21	0.62
100	0.65	0.35	0.85	0.68	0.36	0.86
150	0.80	0.47	0.94	0.84	0.44	0.97

**Scenario C4**				
Effect size	Assign	Survival	Utility (group 1)	Utility (group 2)
Age	0	0	0	0
Sex	0	0	0	0

**Power**						
	Naïve			Propensity matching		
*n*_1_, *n*_2_	*λ*_2_ = 1	*λ*_2_ = 0.5	*λ*_2_ = 2	*λ*_2_ = 1	*λ*_2_ = 0.5	*λ*_2_ = 2
50	0.59	0.28	0.84	0.56	0.28	0.76
100	0.79	0.49	0.97	0.73	0.44	0.94
150	0.92	0.56	0.99	0.90	0.56	0.98

As Table 3 suggests, in Scenario C0, where there is no survival or utility difference between the two treatments, while there are covariates that are affecting the treatment assignment as well as utility scores, the naïve approach that does not adjust for covariates may have highly inflated type I errors. Meanwhile, the propensity matching technique can control type I errors. In Scenario C1, the power of the naïve method may seem much higher, mostly due to its high inflation. In Scenarios C3–C4, the two methods have similar power, though propensity matching may slightly lose power since it uses fewer samples. However, in Scenario C2, where the signs of the covariate effects are modified, the naïve method has very low power, while propensity matching still has decent power. This demonstrates that the confounding issue may also result in loss of power. In conclusion, it is safer to use propensity matching when dealing with observational data, since it can reduce the bias brought by confounders.

### 3.4 Application to Health Utilities Index 3 (HUI3) Data

**FIGURE 3:**
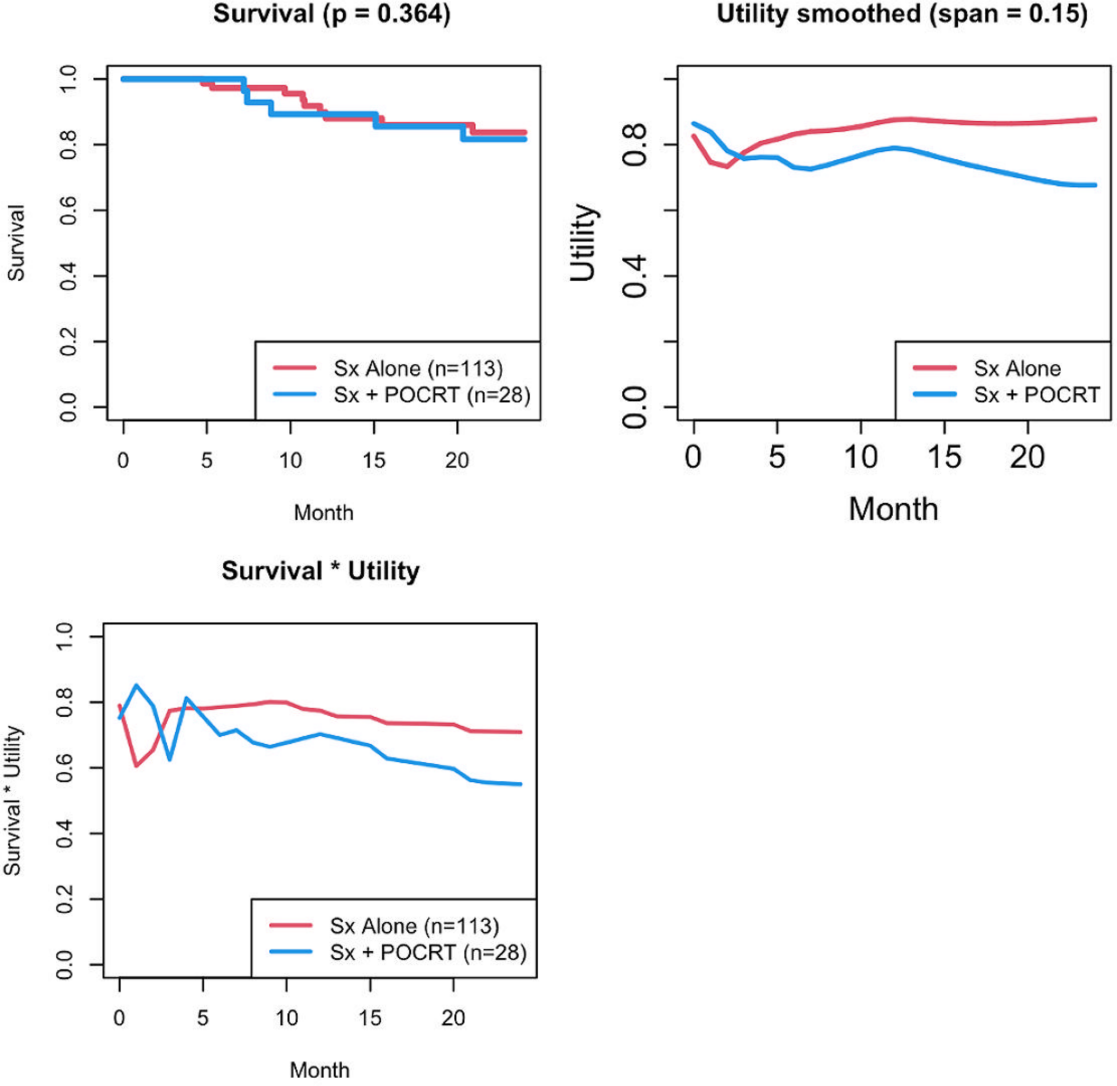
Curves (survival, utility, product of survival and utility) using the group average for the two treatment groups from baseline to 24 months.

To further demonstrate the feasibility of HUS, we apply it to the HUI3 data on a translational patient cohort in Princess Margaret Cancer Centre [34]. It is a retrospective dataset that records the patients' utility scores throughout the study as well as many baseline variables. We are interested in comparing the survival and health utility performance in patients who only received surgery treatments (Sx alone) and patients who received surgery plus combination chemotherapy and radiotherapy (Sx + POCRT). The survival time for patients was recorded from baseline up to 82.4 months, while the utility data was only recorded from baseline to 24 months. The median follow‐up time is 43.8 months. We consider four covariates when conducting propensity matching: age, gender, stage (early or late), and HPV status. Figure 3 shows the sample sizes before and after matching as well as estimated curves based on survival, utility, and the product of survival and utility. We conduct different tests to the data and compare their results.

As shown in Table 4, in this application, the OS‐based log‐rank test has insignificant results, which agrees with the survival plots in Figure 3, where there is no significant difference between the two treatment groups. Note that the *p*‐values in those plots are results using 24 months' survival data only, whereas the *p*‐values of OS in Table 4 use all time‐points (up to 82.4 months). Meanwhile, HUS (except $\lambda_{2}=0.5$) and the test based on utility only are able to obtain significant results, which also agrees with Figure 3. A larger $\lambda_{2}$ leads to a smaller *p*‐value, which makes sense because the difference appears to be in health utility. Giving it a larger weight will lead to more significant results. These test results suggest that the treatment group that receives surgery alone tends to have better utility than the group that receives surgery and postoperative combination chemotherapy and radiotherapy. We also conducted the analysis with propensity matching, however, the sample size left after matching is too small to give meaningful conclusions.

**TABLE 4:** *p*‐values of different tests.

		HUS			
OS	HU only	*λ*_2_ = 1	*λ*_2_ = 0.5	*λ*_2_ = 1.5	*λ*_2_ = 1 (twHUS)
0.513	< 0.0001*	0.048*	0.186	0.016*	0.052

*Note*: OS stands for the log‐rank test to test the difference in overall survival. HU only means compare the health utility without comparing survival (i.e., set $\lambda_{1}=0$, $\lambda_{2}=1$ in HUS).

∗ indicates p < 0.05; significance level was set at 0.05.

## 4 Discussion

We have presented the extensions of HUS to compare treatment effects with a composite endpoint combining survival and health utility with different focuses, and we have established the theoretical properties of HUS. As demonstrated by our comprehensive simulation studies and HUI3 data application, HUS can be applied to not only randomized trial data but also to observational study data, and different versions of HUS show different advantages in various scenarios. The time‐weighted version, twHUS, may yield better results when we care more about the patients' eventual recovery. Using propensity matching is important for observational data as it helps reduce the inflation caused by confounders.

Note that when dealing with multiple QoL measures, the proposed weighted‐average method in this manuscript is straightforward, but there may be other techniques that can boost power in certain scenarios, especially considering there are various choices of measures with different emphases [35, 36, 37]. Exploring other options may be a potential future direction. For instance, we may consider taking a powered sum and applying a technique similar to the SPU (Sum of Powered U‐score) test [38, 39], or building different models with different assumptions and then taking the model average [40]. To better handle sparsely measured utility scores and dependent censoring, we may also consider jointly modeling the scores using a mixed model [41]. Another point worth mentioning is that the choice of weights is important in practice. The main purpose of this manuscript is to further develop the statistical framework of HUS and demonstrate the potentials of its different versions. In practice, we recommend choosing equal weights by default. Based on the clinicians' input, more weights may be given to the measures or time points that are considered more important. On the other hand, if pilot data are available, different choices of weights may be applied based on the prior information and compared before finalizing the choice for the new study. In the analysis stage of the new study, we also recommend conducting sensitivity analyses.

Meanwhile, we have proposed the Cox version of HUS and showed that it has similar performance to using the KM estimates, which is likely due to the fact that our survival data are generated by proportional hazard models. In the future, we may also explore other situations where the proportional hazard assumption is violated and compare the performances of using the Cox estimates and the KM estimates. For example, when the proportional hazards assumption does not hold, the Cox model can be interpreted as estimating a time‐averaged hazard ratio, which may still be quite useful [42, 43]. Besides, it is also possible to perform HUS analysis with other survival models like the flexible parametric model [44], which may show certain benefits in some situations.

Regarding observational study data, there are other options we may compare in terms of handling the confounding issue besides the propensity score matching approach [25, 45, 46]. For example, we may use regression models to estimate and take out the covariate effects, and then use HUS analysis on the residuals. The major challenge about this approach is that it may require very large sample sizes to get good estimates, since when we have many covariates to consider, the number of parameters to estimate in the regression models may be very large. Also, there may be unmeasured confounders that we cannot directly adjust for. Hence, it will be worthwhile to find more efficient and robust ways to analyze observational data with HUS. Besides, though our proposed approach to deal with missing data works well in our simulation settings, where the missingness is independent of or moderately associated with the utility score, we may need to explore better options, given that the missing mechanism in real data may be much less ideal. For example, death may be more related to the utility score. It is important to incorporate more advanced techniques to make HUS more robust in more complicated scenarios [47, 48]. Since this manuscript focuses on the development of statistical properties, the currently used imputation approach is intuitive but relatively simple, which may introduce more bias than some more sophisticated methods. In the future, we may implement more advanced imputation techniques and compare their results [49, 50].

Finally, the current HUS framework is limited to comparing two treatment groups. In certain scenarios, we may have more than two groups of interest, and it may be desirable to have a single test to detect whether there is a difference in multiple groups instead of comparing two groups at a time, which will result in multiple testing and loss of power. For example, in our HUI3 application, we may have interests in comparing three treatment groups: patients who only went through radiation therapy, patients who only received surgery, and patients who received combination treatments. We may develop a multi‐group comparison test based on HUS that can be very useful in these situations.

## 5 Software

R code for our simulation studies is available at https://github.com/yangq001/HUS.

## Conflicts of Interest

The authors declare no conflicts of interest.

## Supporting information


**Data S1.** Supporting information.

**Data S2.** Supporting information.

## Data Availability

The data that support the findings of this study are available from the corresponding author upon reasonable request.
