# Supplementary material for: Health Utility Survival for Randomized Clinical Trials: Extensions and Statistical Properties
Source: Stat Med. 2025 Aug 7;44(18-19):e70215. doi: 10.1002/sim.70215 (PMC12330342; doi:10.1002/sim.70215)
Supplement: Supplementary file 1 — Data S1. Supporting information. [file SIM-44-0-s002.docx]

**Supplementary Material - Health Utility Survival for Randomized Clinical Trials: Extensions and Statistical Properties**

Yangqing Deng^1,*^, Meiling Hao^2,*^, Shao Hui Huang^3^, Geoffrey Liu^4,5,6^, John R. de Almeida^7,8^, Wei Xu^1,6#^

^1^Department of Biostatistics, University Health Network, Toronto, ON, Canada

^2^School of Statistics, University of International Business and Economics, Beijing, China

^3^Department of Radiation Oncology, University Health Network, Toronto, ON, Canada

^4^Temerty Faculty of Medicine, University of Toronto, Toronto, ON, Canada

^5^Medical Oncology and Hematology, Princess Margaret Cancer Centre, Toronto, ON, Canada

^6^Dalla Lana School of Public Health, University of Toronto, Toronto, ON, Canada

^7^Department of Otolaryngology—H&N Surgery, University Health Network, Toronto, ON, Canada

^8^Institute of Health Policy, Management and Evaluation, University of Toronto, Toronto, ON, Canada

^*^Co-first-authors

^#^Corresponding author: Wei Xu, Email: wei.xu@uhnresearch.ca

# Appendix A, Additional Details on Theoretical Properties

## Proof of Lemma 1

$$\sqrt{n_{1}}\left\{ \hat{S}_{1}\left( t \right)\bar{U}_{1}\left( t \right)-S_{1}\left( t \right)U_{1}\left( t \right) \right\}$$

$$=\sqrt{n_{1}}\left\{ \hat{S}_{1}\left( t \right)\bar{U}_{1}\left( t \right)-S_{1}\left( t \right)\bar{U}_{1}\left( t \right) \right\}+\sqrt{n_{1}}S_{1}\left( t \right)\left\{ \bar{U}_{1}\left( t \right)-U_{1}\left( t \right) \right\}$$

$$=-\left[ \sqrt{n_{1}}S_{1}\left( t \right)\sum_{i=1}^{n_{1}} \int_{0}^{t} \frac{dM_{1i}\left( u \right)}{{\bar{\mathbb{Y}}}_{1}\left( u \right)}+o_{p}\left( 1 \right) \right]\bar{U}_{1}\left( t \right)+\sqrt{n_{1}}S_{1}\left( t \right)\left\{ \bar{U}_{1}\left( t \right)-U_{1}\left( t \right) \right\}$$

$$=\mathcal{G}_{1}\left( t \right)U_{1}\left( t \right)+S_{1}\left( t \right)\mathcal{U}_{1}\left( t \right)+o_{p}\left( 1 \right).$$

The second equality follows (Fleming and Harrington 2005), where $M_{1i}\left( t \right)=N_{1i}\left( t \right)-\int_{0}^{t} Y_{1i}\left( u \right)\lambda(u)du$. Note that due to right censoring, this approximation is only valid for the interval $t\in[0,T]$, where $P\left( X_{1i}>T \right)>0$. It follows from the martingale central limit theorem (Fleming and Harrington 2005) that $\sqrt{n_{1}}\left\{ \hat{S}_{1}\left( t \right)-S_{1}\left( t \right) \right\}$ converges weakly to a zero mean Gaussian process $\mathcal{G}_{1}\left( t \right)$ over the interval $[0,T]$. Furthermore, since $U_{1i}\left( t \right)$ is of bounded variation on $[0,T]$, it follows the central limit theorem (Pollard 1990) that implies $\sqrt{n_{1}}\left\{ \bar{U}_{1}\left( t \right)-U_{1}\left( t \right) \right\}$ converges to a zero mean Gaussian process $\mathcal{U}_{1}\left( t \right)$ over the interval $[0,T]$.

## Proof of Theorem 1

$$\sqrt{n_{1}}\hat{\mathcal{T}}=\sqrt{n}_{1}\left( \hat{Q}_{HUS,1}-\hat{Q}_{HUS,2} \right)$$

$$=\sqrt{n}_{1}\int_{0}^{T} \left\{ \hat{S}_{1}\left( t \right)\bar{U}_{1}\left( t \right)-\hat{S}_{2}\left( t \right)\bar{U}_{2}\left( t \right) \right\}dt$$

$$=\sqrt{n_{1}}\int_{0}^{T} \left\{ \hat{S}_{1}\left( t \right)\bar{U}_{1}\left( t \right)-S_{1}\left( t \right)U_{1}\left( t \right)+S_{1}\left( t \right)U_{1}\left( t \right)-\hat{S}_{2}\left( t \right)\bar{U}_{2}\left( t \right) \right\}dt$$

$$=\sqrt{n}_{1}\int_{0}^{T} \left\{ \hat{S}_{1}\left( t \right)\bar{U}_{1}\left( t \right)-S_{1}\left( t \right)U_{1}\left( t \right) \right\}dt-\frac{\sqrt{n_{1}}}{\sqrt{n_{2}}}\sqrt{n_{2}}\int_{0}^{T} \left\{ \hat{S}_{2}\left( t \right)\bar{U}_{2}\left( t \right)-S_{1}\left( t \right)U_{1}\left( t \right) \right\}dt$$

$$=\sqrt{1+c}\int_{0}^{T} \mathcal{G}_{1}(t)U_{1}\left( t \right)+S_{1}\left( t \right)\mathcal{U}_{1}\left( t \right)dt+o_{p}\left( 1 \right)$$

The last equality follows from Lemma 1, the functional δ-method and the property of normal distribution.

## Proof of Lemma 2

If follows from similar arguments at that in the proof of Lemma 1 and the functional delta method that

$$\sqrt{n_{1}}\left\{ \left[ \hat{S}_{1}\left( t \right) \right]^{\lambda_{1}}\left[ \bar{U}_{1}\left( t \right) \right]^{\lambda_{2}}-\left[ S_{1}\left( t \right) \right]^{\lambda_{1}}\left[ U_{1}\left( t \right) \right]^{\lambda_{2}} \right\}$$

$$=\sqrt{n_{1}}\left\{ \left[ \hat{S}_{1}\left( t \right) \right]^{\lambda_{1}}\left[ \bar{U}_{1}\left( t \right) \right]^{\lambda_{2}}-\left[ S_{1}\left( t \right) \right]^{\lambda_{1}}\left[ \bar{U}_{1}\left( t \right) \right]^{\lambda_{2}} \right\}+\sqrt{n_{1}}\left[ \hat{S}_{1}\left( t \right) \right]^{\lambda_{1}}\left\{ \left[ \bar{U}_{1}\left( t \right) \right]^{\lambda_{2}}-\left[ U_{1}\left( t \right) \right]^{\lambda_{2}} \right\}$$

$$=-\lambda_{1}\left[ S_{1}\left( t \right) \right]^{\lambda_{1}-1}\left\{ \sqrt{n_{1}}\left[ S_{1}\left( t \right) \right]\sum_{i=1}^{n_{1}} \int_{0}^{t} \frac{dM_{1i}\left( u \right)}{{\bar{\mathbb{Y}}}_{1}\left( u \right)}+o_{p}\left( 1 \right) \right\}\left[ \bar{U}_{1}\left( t \right) \right]^{\lambda_{2}}+\lambda_{2}\sqrt{n_{1}}\left[ S_{1}\left( t \right) \right]^{\lambda_{1}}\left[ U_{1}\left( t \right) \right]^{\lambda_{2}-1}\left\{ \bar{U}_{1}\left( t \right)-U_{1}\left( t \right) \right\}+o_{p}(1)$$

$$=\lambda_{1}\mathcal{G}_{1}\left( t \right)\left[ S_{1}\left( t \right) \right]^{\lambda_{1}-1}\left[ U_{1}\left( t \right) \right]^{\lambda_{2}}+\lambda_{2}\left[ S_{1}\left( t \right) \right]^{\lambda_{1}}\left[ U_{1}\left( t \right) \right]^{\lambda_{2}-1}\mathcal{U}_{1}\left( t \right)+o_{p}\left( 1 \right).$$

## Proof of Theorem 2

$$\sqrt{n}_{1}\hat{\mathcal{T}}=\sqrt{n}_{1}\left( \hat{Q}_{HUS,1}-\hat{Q}_{HUS,2} \right)$$

$$=\sqrt{n}\int_{0}^{\tau} \left\{ \left[ \hat{S}_{1}\left( t \right) \right]^{\lambda_{1}}\left[ \bar{U}_{1}\left( t \right) \right]^{\lambda_{2}}-\left[ \hat{S}_{2}\left( t \right) \right]^{\lambda_{1}}\left[ \bar{U}_{2}\left( t \right) \right]^{\lambda_{2}} \right\}dt$$

$$=\sqrt{n}_{1}\int_{0}^{\tau} \left\{ \left[ \hat{S}_{1}\left( t \right) \right]^{\lambda_{1}}\left[ \bar{U}_{1}\left( t \right) \right]^{\lambda_{2}}-\left[ S_{1}\left( t \right) \right]^{\lambda_{1}}\left[ U_{1}\left( t \right) \right]^{\lambda_{2}}+\left[ S_{1}\left( t \right) \right]^{\lambda_{1}}\left[ U_{1}\left( t \right) \right]^{\lambda_{2}}-\left[ \hat{S}_{2}\left( t \right) \right]^{\lambda_{1}}\left[ \bar{U}_{2}\left( t \right) \right]^{\lambda_{2}} \right\}dt$$

$$=\sqrt{n_{1}}\int_{0}^{\tau} \left\{ \left[ \hat{S}_{1}\left( t \right) \right]^{\lambda_{1}}\left[ \bar{U}_{1}\left( t \right) \right]^{\lambda_{2}}-\left[ S_{1}\left( t \right) \right]^{\lambda_{1}}\left[ U_{1}\left( t \right) \right]^{\lambda_{2}} \right\}dt-\frac{\sqrt{n_{1}}}{\sqrt{n}_{2}}\sqrt{n_{2}}\int_{0}^{\tau} \left\{ \left[ \hat{S}_{2}\left( t \right) \right]^{\lambda_{1}}\left[ \bar{U}_{2}\left( t \right) \right]^{\lambda_{2}}-\left[ S_{1}\left( t \right) \right]^{\lambda_{1}}\left[ U_{1}\left( t \right) \right]^{\lambda_{2}} \right\}dt$$

$$=\sqrt{1+c}\int_{0}^{T} \lambda_{1}\mathcal{G}_{1}\left( t \right)\left[ S_{1}\left( t \right) \right]^{\lambda_{1}-1}\left[ U_{1}\left( t \right) \right]^{\lambda_{2}}+\lambda_{2}\left[ S_{1}\left( t \right) \right]^{\lambda_{1}}\left[ U_{1}\left( t \right) \right]^{\lambda_{2}-1}\mathcal{U}_{1}\left( t \right)dt+o_{p}\left( 1 \right).$$

The last equality follows from Lemma 2, the functional δ-method and the property of normal distribution.

## Proof of Lemma 3

It follows from similar arguments as that in Lemma 2 that

$$\sqrt{n_{1}}\left\{ \left[ \hat{S}_{1}\left( t \right) \right]^{\lambda_{1}}\left[ w\left( t \right)\bar{U}_{1}\left( t \right) \right]^{\lambda_{2}}-\left[ S_{1}\left( t \right) \right]^{\lambda_{1}}\left[ w\left( t \right)U_{1}\left( t \right) \right]^{\lambda_{2}} \right\}$$

$$=\sqrt{n_{1}}\left\{ \left[ \hat{S}_{1}\left( t \right) \right]^{\lambda_{1}}\left[ w\left( t \right)\bar{U}_{1}\left( t \right) \right]^{\lambda_{2}}-\left[ S_{1}\left( t \right) \right]^{\lambda_{1}}\left[ w\left( t \right)\bar{U}_{1}\left( t \right) \right]^{\lambda_{2}} \right\}+\sqrt{n_{1}}\left[ \hat{S}_{1}\left( t \right) \right]^{\lambda_{1}}\left\{ \left[ w\left( t \right)\bar{U}_{1}\left( t \right) \right]^{\lambda_{2}}-\left[ w\left( t \right)U_{1}\left( t \right) \right]^{\lambda_{2}} \right\}$$

$$=-\lambda_{1}\left[ S_{1}\left( t \right) \right]^{\lambda_{1}-1}\left\{ \sqrt{n_{1}}\left[ S_{1}\left( t \right) \right]\sum_{i=1}^{n_{1}} \int_{0}^{t} \frac{dM_{1i}\left( u \right)}{{\bar{\mathbb{Y}}}_{1}\left( u \right)}+o_{p}\left( 1 \right) \right\}\left[ w\left( t \right)\bar{U}_{1}\left( t \right) \right]^{\lambda_{2}}+\lambda_{2}\sqrt{n_{1}}\left[ S_{1}\left( t \right) \right]^{\lambda_{1}}\left[ w\left( t \right) \right]^{\lambda_{2}}\left[ U_{1}\left( t \right) \right]^{\lambda_{2}-1}\left\{ \bar{U}_{1}\left( t \right)-U_{1}\left( t \right) \right\}+o_{p}(1)$$

$$=\lambda_{1}\mathcal{G}_{1}(t){[S_{1}\left( t \right)]}^{\lambda_{1}-1}{[w(t)U_{1}\left( t \right)]}^{\lambda_{2}}+\lambda_{2}{[S_{1}\left( t \right)]}^{\lambda_{1}}{[w(t)]}^{\lambda_{2}}{[U_{1}\left( t \right)]}^{\lambda_{2}-1}\mathcal{U}_{1}\left( t \right)+o_{p}\left( 1 \right).$$

## Proof of Theorem 3

$$\sqrt{n_{1}}{\hat{\mathcal{T}}}_{t}=\sqrt{n}\left( \hat{Q}_{tHUS,1}-\hat{Q}_{tHUS,2} \right)$$

$$=\sqrt{n}_{1}\int_{0}^{T} \left\{ \left[ \hat{S}_{1}\left( t \right) \right]^{\lambda_{1}}\left[ w\left( t \right)\bar{U}_{1}\left( t \right) \right]^{\lambda_{2}}-\left[ \hat{S}_{2}\left( t \right) \right]^{\lambda_{1}}\left[ w\left( t \right)\bar{U}_{2}\left( t \right) \right]^{\lambda_{2}} \right\}dt$$

$$=\sqrt{n_{1}}\int_{0}^{T} \left\{ \left[ \hat{S}_{1}\left( t \right) \right]^{\lambda_{1}}\left[ w\left( t \right)\bar{U}_{1}\left( t \right) \right]^{\lambda_{2}}-\left[ S_{1}\left( t \right) \right]^{\lambda_{1}}\left[ w\left( t \right)U_{1}\left( t \right) \right]^{\lambda_{2}}+\left[ S_{1}\left( t \right) \right]^{\lambda_{1}}\left[ w\left( t \right)U_{1}\left( t \right) \right]^{\lambda_{2}}-\left[ \hat{S}_{2}\left( t \right) \right]^{\lambda_{1}}\left[ w\left( t \right)\bar{U}_{2}\left( t \right) \right]^{\lambda_{2}} \right\}dt$$

$$=\sqrt{n_{1}}\int_{0}^{T} \left\{ \left[ \hat{S}_{1}\left( t \right) \right]^{\lambda_{1}}\left[ w\left( t \right)\bar{U}_{1}\left( t \right) \right]^{\lambda_{2}}-\left[ S_{1}\left( t \right) \right]^{\lambda_{1}}\left[ w\left( t \right)U_{1}\left( t \right) \right]^{\lambda_{2}} \right\}dt-\int_{0}^{T} \left\{ \left[ \hat{S}_{2}\left( t \right) \right]^{\lambda_{1}}\left[ w\left( t \right)\bar{U}_{2}\left( t \right) \right]^{\lambda_{2}}-\left[ S_{1}\left( t \right) \right]^{\lambda_{1}}\left[ w\left( t \right)U_{1}\left( t \right) \right]^{\lambda_{2}} \right\}dt$$

$$=\sqrt{1+c}\int_{0}^{T} \lambda_{1}\mathcal{G}_{1}\left( t \right)\left[ S_{1}\left( t \right) \right]^{\lambda_{1}-1}\left[ w\left( t \right)U_{1}\left( t \right) \right]^{\lambda_{2}}+\lambda_{2}\left[ S_{1}\left( t \right) \right]^{\lambda_{1}}{[w(t)]}^{\lambda_{2}}\left[ U_{1}\left( t \right) \right]^{\lambda_{2}-1}\mathcal{U}_{1}\left( t \right)dt+o_{p}\left( 1 \right).$$

The last equality follows from Lemma 3, the functional δ-method and the property of normal distribution.

# Appendix B, Additional Simulation Results

## Comparison of Imputation Methods

As examples to demonstrate the effectiveness of our newly proposed imputation method, we apply both imputation methods mentioned in section 2.6 to Scenarios A1 and B1 in the main article. For imputation method 1, we apply linear imputation to each subject without using other subjects’ scores. For imputation method 2, we first impute the missing scores using the group average at time-points where at least 80% of the subjects have their scores recorded, and then use linear imputation to fill in the rest of the missing values. According to Table S1, imputation method 2 has higher power, which is why we use this method by default in the main article.

**Table S1.** Comparison of the two imputation methods (with or without imputing the group average).

| Scenario A1 | | | | | | | | |
| --- | --- | --- | --- | --- | --- | --- | --- | --- |
| $n_{1}, n_{2}$ | KM (imputation method 1) | | | | KM (imputation method 2) | | | |
|  | $\lambda_{2}=1$ | $\lambda_{2}=0.5$ | $\lambda_{2}=2$ | $\lambda_{2}=1$ (twHUS) | $\lambda_{2}=1$ | $\lambda_{2}=0.5$ | $\lambda_{2}=2$ | $\lambda_{2}=1$ (twHUS) |
| 50 | 0.59 | 0.32 | 0.83 | 0.58 | 0.81 | 0.41 | 0.99 | 0.8 |
| 100 | 0.73 | 0.48 | 0.90 | 0.71 | 0.95 | 0.62 | 1 | 0.95 |
| 150 | 0.92 | 0.60 | 0.98 | 0.92 | 0.99 | 0.78 | 1 | 0.99 |
| $n_{1}, n_{2}$ | Cox (imputation method 1) | | | | Cox (imputation method 2) | | | |
|  | $\lambda_{2}=1$ | $\lambda_{2}=0.5$ | $\lambda_{2}=2$ | $\lambda_{2}=1$ (twHUS) | $\lambda_{2}=1$ | $\lambda_{2}=0.5$ | $\lambda_{2}=2$ | $\lambda_{2}=1$ (twHUS) |
| 50 | 0.66 | 0.37 | 0.84 | 0.66 | 0.85 | 0.46 | 1 | 0.85 |
| 100 | 0.75 | 0.50 | 0.90 | 0.73 | 0.98 | 0.7 | 1 | 0.98 |
| 150 | 0.94 | 0.68 | 0.98 | 0.94 | 1 | 0.86 | 1 | 1 |
| Scenario B1 | | | | | | | | |
| $n_{1}, n_{2}$ | KM (imputation method 1) | | | | KM (imputation method 2) | | | |
|  | $\lambda_{2}=1$ | $\lambda_{2}=0.5$ | $\lambda_{2}=2$ | $\lambda_{2}=1$ (twHUS) | $\lambda_{2}=1$ | $\lambda_{2}=0.5$ | $\lambda_{2}=2$ | $\lambda_{2}=1$ (twHUS) |
| 50 | 0.50 | 0.22 | 0.80 | 0.46 | 0.74 | 0.32 | 0.97 | 0.72 |
| 100 | 0.68 | 0.40 | 0.89 | 0.66 | 0.9 | 0.57 | 1 | 0.9 |
| 150 | 0.86 | 0.51 | 0.98 | 0.84 | 0.99 | 0.67 | 1 | 0.98 |
| $n_{1}, n_{2}$ | Cox (imputation method 1) | | | | Cox (imputation method 2) | | | |
|  | $\lambda_{2}=1$ | $\lambda_{2}=0.5$ | $\lambda_{2}=2$ | $\lambda_{2}=1$ (twHUS) | $\lambda_{2}=1$ | $\lambda_{2}=0.5$ | $\lambda_{2}=2$ | $\lambda_{2}=1$ (twHUS) |
| 50 | 0.52 | 0.31 | 0.80 | 0.50 | 0.8 | 0.39 | 0.98 | 0.78 |
| 100 | 0.69 | 0.44 | 0.91 | 0.69 | 0.92 | 0.6 | 1 | 0.92 |
| 150 | 0.90 | 0.59 | 0.98 | 0.90 | 0.99 | 0.76 | 1 | 0.98 |

## Comparison of Bootstrap and Perturbation Resampling

We compare the bootstrap method, which we use by default in the main article, with the perturbation-resampling method using Scenarios A0-A1 and B1-B2. As shown in Table S2, the two approaches have similar results. We recommend using the bootstrap approach by default due to its simplicity.

**Table S2.** Comparison of the two resampling methods using KM estimates for survival.

| Scenario A0 | | | | | | | | |
| --- | --- | --- | --- | --- | --- | --- | --- | --- |
| $n_{1}, n_{2}$ | Bootstrap | | | | Perturbation resampling | | | |
|  | $\lambda_{2}=1$ | $\lambda_{2}=0.5$ | $\lambda_{2}=2$ | $\lambda_{2}=1$ (twHUS) | $\lambda_{2}=1$ | $\lambda_{2}=0.5$ | $\lambda_{2}=2$ | $\lambda_{2}=1$ (twHUS) |
| 50 | 0.048 | 0.047 | 0.053 | 0.048 | 0.051 | 0.050 | 0.048 | 0.048 |
| 100 | 0.046 | 0.045 | 0.051 | 0.046 | 0.050 | 0.051 | 0.051 | 0.051 |
| 150 | 0.054 | 0.054 | 0.052 | 0.050 | 0.050 | 0.046 | 0.050 | 0.052 |
| Scenario A1 | | | | | | | | |
| $n_{1}, n_{2}$ | Bootstrap | | | | Perturbation resampling | | | |
|  | $\lambda_{2}=1$ | $\lambda_{2}=0.5$ | $\lambda_{2}=2$ | $\lambda_{2}=1$ (twHUS) | $\lambda_{2}=1$ | $\lambda_{2}=0.5$ | $\lambda_{2}=2$ | $\lambda_{2}=1$ (twHUS) |
| 50 | 0.81 | 0.41 | 0.99 | 0.80 | 0.82 | 0.40 | 1 | 0.79 |
| 100 | 0.95 | 0.62 | 1 | 0.95 | 0.93 | 0.64 | 1 | 0.92 |
| 150 | 0.99 | 0.78 | 1 | 0.99 | 0.98 | 0.78 | 1 | 0.99 |
| Scenario B1 | | | | | | | | |
| $n_{1}, n_{2}$ | Bootstrap | | | | Perturbation resampling | | | |
|  | $\lambda_{2}=1$ | $\lambda_{2}=0.5$ | $\lambda_{2}=2$ | $\lambda_{2}=1$ (twHUS) | $\lambda_{2}=1$ | $\lambda_{2}=0.5$ | $\lambda_{2}=2$ | $\lambda_{2}=1$ (twHUS) |
| 50 | 0.74 | 0.32 | 0.97 | 0.72 | 0.72 | 0.31 | 0.98 | 0.72 |
| 100 | 0.90 | 0.57 | 1 | 0.90 | 0.89 | 0.55 | 1 | 0.89 |
| 150 | 0.99 | 0.67 | 1 | 0.98 | 1 | 0.68 | 1 | 0.98 |
| Scenario B2 | | | | | | | | |
| $n_{1}, n_{2}$ | Bootstrap | | | | Perturbation resampling | | | |
|  | $\lambda_{2}=1$ | $\lambda_{2}=0.5$ | $\lambda_{2}=2$ | $\lambda_{2}=1$ (twHUS) | $\lambda_{2}=1$ | $\lambda_{2}=0.5$ | $\lambda_{2}=2$ | $\lambda_{2}=1$ (twHUS) |
| 50 | 0.24 | 0.11 | 0.71 | 0.58 | 0.25 | 0.12 | 0.73 | 0.56 |
| 100 | 0.44 | 0.17 | 0.9 | 0.82 | 0.43 | 0.16 | 0.89 | 0.81 |
| 150 | 0.54 | 0.21 | 0.99 | 0.92 | 0.53 | 0.21 | 0.96 | 0.93 |

## Simulation Results with Informative Missing

We examine the performance of HUS under two settings of informative missing. First, we generate the data following Scenarios A0, A1, B1, B2 in section 3.1 of the main article. Then, in setting I, we specify that if the utility of a subject at any timepoint is less than 0.35, then their utility at later timepoints will all be missing (about 12% of the utility scores generated in Scenario A0 satisfy this criterion). In setting II, we specify that if the simulated utility of a subject at a timepoint is less than 0.6, then there is an additional 50% chance for this utility to be missing (about 35% of the utility scores generated in Scenario A0 satisfy this criterion). As a result, in either setting, the missingness of utility is associated with its value. According to Table S3, HUS has relatively good performance under moderate informative missing, with slightly reduced power.

**Table S3.** HUS performance under moderate informative missing. The bootstrap method is used by default.

| Scenario A0 | | | | | | | | |
| --- | --- | --- | --- | --- | --- | --- | --- | --- |
| $n_{1}, n_{2}$ | Setting I | | | | Setting II | | | |
|  | $\lambda_{2}=1$ | $\lambda_{2}=0.5$ | $\lambda_{2}=2$ | $\lambda_{2}=1$ (twHUS) | $\lambda_{2}=1$ | $\lambda_{2}=0.5$ | $\lambda_{2}=2$ | $\lambda_{2}=1$ (twHUS) |
| 50 | 0.053 | 0.054 | 0.055 | 0.055 | 0.056 | 0.047 | 0.056 | 0.053 |
| 150 | 0.053 | 0.051 | 0.054 | 0.051 | 0.054 | 0.053 | 0.055 | 0.053 |
| Scenario A1 | | | | | | | | |
| $n_{1}, n_{2}$ | Setting I | | | | Setting II | | | |
|  | $\lambda_{2}=1$ | $\lambda_{2}=0.5$ | $\lambda_{2}=2$ | $\lambda_{2}=1$ (twHUS) | $\lambda_{2}=1$ | $\lambda_{2}=0.5$ | $\lambda_{2}=2$ | $\lambda_{2}=1$ (twHUS) |
| 50 | 0.74 | 0.40 | 0.96 | 0.74 | 0.78 | 0.40 | 0.98 | 0.77 |
| 150 | 0.98 | 0.76 | 1 | 0.98 | 0.98 | 0.78 | 1 | 0.98 |
| Scenario B1 | | | | | | | | |
| $n_{1}, n_{2}$ | Setting I | | | | Setting II | | | |
|  | $\lambda_{2}=1$ | $\lambda_{2}=0.5$ | $\lambda_{2}=2$ | $\lambda_{2}=1$ (twHUS) | $\lambda_{2}=1$ | $\lambda_{2}=0.5$ | $\lambda_{2}=2$ | $\lambda_{2}=1$ (twHUS) |
| 50 | 0.68 | 0.3 | 0.96 | 0.65 | 0.66 | 0.28 | 0.94 | 0.64 |
| 150 | 0.98 | 0.67 | 1 | 0.98 | 0.98 | 0.66 | 1 | 0.97 |
| Scenario B2 | | | | | | | | |
| $n_{1}, n_{2}$ | Setting I | | | | Setting II | | | |
|  | $\lambda_{2}=1$ | $\lambda_{2}=0.5$ | $\lambda_{2}=2$ | $\lambda_{2}=1$ (twHUS) | $\lambda_{2}=1$ | $\lambda_{2}=0.5$ | $\lambda_{2}=2$ | $\lambda_{2}=1$ (twHUS) |
| 50 | 0.22 | 0.07 | 0.69 | 0.54 | 0.23 | 0.08 | 0.7 | 0.54 |
| 150 | 0.51 | 0.18 | 0.98 | 0.92 | 0.54 | 0.19 | 0.98 | 0.91 |
